# Clinical Cases

**Published:** 1883-06

**Authors:** J. W. Thomson

**Affiliations:** New Haven, Conn.


### CLINICAL CASE.

J. W. Thomson, M. D., New Haven, Conn.

H. J. M., boy, æt. 5½ years. Light complexion and stout, but looks flabby, and flesh has a doughy feel. Cannot hold his urine day or night, or even wait to ease himself, and has to wear a cloth continuously. Legs raw and sore from constant irritation of urine, which is so strong and offensive that the boy is in constant misery. The stains on his clothing are of a deep yellow and brown color; the smell is very offensive. When he happens to be able to use a vessel, which is very seldom, so sudden is the neceesity, there is a red sediment which adheres thereto. He urinates at least twenty times a day.

Has a dry cough with rattling in chest; he never raises at all. Don't cough if he keeps quiet, but the least exertion provokes it, and also causes him to turn pale. He delights to be left alone. Says his head is tired.

Dr. D., the leading allopath, his mother informed me, stated that her husband died of "heart disease." She said that she had doctored and doctored and doctored her boy, but it was all of no use. Not anything that he had ever taken had helped him in the least. She had often punished him for wetting the bed and his clothing, but was now satisfied that he could not help it, and therefore spared the rod. Had given up all hope of getting help for him. But having heard of cases that I had cured she brought him to me. ℞. Benz-ac.[200] (D.) A powder to be given at 11 o'clock A. M., daily, for six days and then report.

When I again saw the woman she stated that on the following night, to her utter astonishment, her boy did not wet the bed. Such an omission had hardly ever occurred before. Next morning he urinated most profusely. He had never passed so much at a time. Ever since he had had perfect control of his urine. He had been out to play frequently and had wet feet, but he could hold his urine now. He had no cough, did not complain of his head, or want to be alone so much as formerly.

Although the result was apparently all that could be desired, yet I have often wished I had only given a single dose of the remedy, because, as I was informed, there had been no mictio involuntaria on the night following, and the bladder was so tolerant of its contents as to be full to repletion. However, mother and son were both satisfied.

